# α-Tocopherol at Nanomolar Concentration Protects PC12 Cells from Hydrogen Peroxide-Induced Death and Modulates Protein Kinase Activities

**DOI:** 10.3390/ijms130911543

**Published:** 2012-09-14

**Authors:** Irina O. Zakharova, Tatyana V. Sokolova, Liubov V. Bayunova, Yulia A. Vlasova, Maria P. Rychkova, Natalia F. Avrova

**Affiliations:** Department of Comparative Neurochemistry, Institute of Evolutionary Physiology and Biochemistry of Russian Academy of Sciences, Thorez avenue, 44, Saint-Petersburg 194223, Russia; E-Mails: zakhar@iephb.ru (I.O.Z.); sokolt1956@mail.ru (T.V.S.); bayunoval@mail.ru (L.V.B.); yousia@mail.ru (Y.A.V.); involved@mail.ru (M.P.R.)

**Keywords:** α-tocopherol, physiological concentrations, PC12 cells, oxidative stress, anti-apoptotic compound, MEK/ERK signaling pathway, Akt

## Abstract

The aim of this work was to compare protective and anti-apoptotic effects of α-tocopherol at nanomolar and micromolar concentrations against 0.2 mM H_2_O_2_-induced toxicity in the PC12 neuronal cell line and to reveal protein kinases that contribute to α-tocopherol protective action. The protection by 100 nM α-tocopherol against H_2_O_2_-induced PC12 cell death was pronounced if the time of pre-incubation with α-tocopherol was 3–18 h. For the first time, the protective effect of α-tocopherol was shown to depend on its concentration in the nanomolar range (1 nM < 10 nM < 100 nM), if the pre-incubation time was 18 h. Nanomolar and micromolar α-tocopherol decreased the number of PC12 cells in late apoptosis induced by H_2_O_2_ to the same extent if pre-incubation time was 18 h. Immunoblotting data showed that α-tocopherol markedly diminished the time of maximal activation of extracellular signal-regulated kinase 1/2 (ERK 1/2) and protein kinase B (Akt)-induced in PC12 cells by H_2_O_2_. Inhibitors of MEK 1/2, PI 3-kinase and protein kinase C (PKC) diminished the protective effect of α-tocopherol against H_2_O_2_-initiated toxicity if the pre-incubation time was long. The modulation of ERK 1/2, Akt and PKC activities appears to participate in the protection by α-tocopherol against H_2_O_2_-induced death of PC12 cells. The data obtained suggest that inhibition by α-tocopherol in late stage ERK 1/2 and Akt activation induced by H_2_O_2_ in PC12 cells makes contribution to its protective effect, while total inhibition of these enzymes is not protective.

## 1. Introduction

α-Tocopherol (α-T) is the main vitamin E component of various human and animal organs. Specific α-T transfer and binding proteins prevent it from metabolic degradation. Various vitamin E isoforms (α-, β-, γ- and δ-tocopherols and tocotrienols) possess radical scavenging activity and modulate activity of signaling pathways. α-T was shown to inhibit protein kinase C (PKC), phosphatidyl inositol 3-kinase (PI 3-kinase), and to activate protein phosphatases, especially protein phosphatase 2A and lipid phosphatases, for example, PTEN. In addition, evidence was obtained that α-T modulated the activity of cycloxygenase 1/2, lipoxygenases, NADPH oxidase and the function of ion channels [1–8].

There is a point of view that α-T is an “antioxidant and nothing more” [9]. However, the inability of other vitamin E components to substitute for α-T in a number of cellular reactions (although various vitamin E components have the similar radical scavenging activity) and other facts provide evidence of non-antioxidant molecular functions of α-T [3,4]. In the brain, α-T functions and effects appear not to depend only on its radical scavenging activity. Thus, the long-term (up to 46 weeks) maintenance of mice on a vitamin E-deficient diet and the use of the mice with targeted disruption of the gene for α-T transfer protein (mutations in this gene lead to hereditary ataxia with vitamin E deficiency in humans) resulted in a strong decrease of α-T content in the brain and other organs [10]. However, the intensity of peroxidation of murine brain lipids was not increased. On the contrary, it was markedly diminished [10].

In the central nervous system, α-T is present in the cerebrospinal fluid as well as in the extracellular brain space at nanomolar concentrations, as delivery exists from blood to cerebrospinal fluid across the blood-brain barrier. The concentration of α-T was estimated to be 42.1 ± 17.0 nM in human cerebrospinal fluid, while the concentration of other vitamin E components is much lower, e.g., γ-tocopherol concentration was found to be 5.9 ± 2.8 nM [11]. This is consistent with data of other earlier studies [12,13].

It is of interest to find out if α-T and other vitamin E components at their physiological (nanomolar) concentration are able to protect cultured neurons and cells of neuronal cell lines from toxin-induced death. Interesting and informative data were obtained on the mechanism of protective effect of nanomolar α-tocotrienol against glutamate-induced toxicity in the cultured neurons and neuronal cell line. This effect was shown to depend on inhibition of 12-lipoxygenase and phospholipase A_2_ by α-tocotrienol [14,15], α-tocotrienol at nanomolar concentration or inhibitors of 12-lipoxygenase increased the viability of both cultured hippocampal neurons, and cells of the HT4 hippocampal cell line exposed to glutamate. However, nanomolar α-T was shown not to be protective under such experimental conditions [14–16].

Studies of the mechanism of the α-T protective effect against the oxidative stress-induced death of cultured neurons and cells of neuronal cell lines are scarce [1,7,17,18]. The results of these studies suggest that cultured neurons and neuronal cell lines are sensitive to the effect of α-T at its physiological nanomolar concentration. Thus, nanomolar α-T was shown to protect primary cultures of nerve cells [17] or cells of neuronal cell lines [18] from death induced by toxins which caused the activation of free radical reactions in these cells. The long pre-treatment of hippocampal neurons with rather low micromolar concentrations of α-T (1–2.5 μM) prior to the induction of oxidative stress by Fe^2+^ [1,7] was also shown to provide a long-lasting protection via genomic activation, which was in contrast to the transient effect of 10 μM α-T, based on its radical scavenging activity.

Numakawa and co-authors [17] showed that a prolonged pre-incubation of cultured immature cortical neurons with α-T at nanomolar concentrations enhanced the survival of these cells when they were subsequently exposed to H_2_O_2_. Nanomolar α-T was shown to increase the activity of the basal extracellular signal-regulated kinase 1/2 (ERK 1/2) and protein kinase B (Akt) in the cortical neurons [17]. However, in this work, it was neither shown in what range of concentrations the effect of α-T was concentration-dependent, nor were the data provided on protein kinase activities under the conditions of oxidative stress.

Since nanomolar concentrations of α-T and other vitamin E components are characteristic for cerebrospinal fluid and for brain extracellular space of humans and animals [11–13], it seems of importance to understand the mechanism of the metabolic and protective effects of these compounds at physiological concentrations, as their effects may differ from the effects of these compounds in pharmacological concentrations. Thus, one of the reviews devoted to modulation of signal transduction by vitamin E components in the cells of various organs ends with the following conclusion: “An important issue to be addressed in the future will be to evaluate which of these regulatory effects represent physiological events responsible for the essentiality of vitamin E, and which of them rather correspond to pharmacological effects of vitamin E analogues that normally do not reach a high *in vivo* concentration” [4].

In this context, the aims of the present work were to find out if α-T at nanomolar concentration had a protective effect on a PC12 neuronal cell line exposed to H_2_O_2_, to reveal how the protective and anti-apoptotic effect of α-T depended on its concentration at short and long periods of pre-incubation, and to assess the contribution of modulation of ERK 1/2, Akt and PKC activities by nanomolar and micromolar α-T under conditions of oxidative stress to its protective effect on PC12 cells. The protective effect of nanomolar α-T against hydrogen peroxide-induced death of PC12 cells and immature cortical neurons was found to be similar to the effect of micromolar α-T if pre-incubation with α-T was performed for 18 h. α-T was found to decrease markedly the time of maximal activation of ERK 1/2 and Akt induced in PC12 cells by H_2_O_2_.

## 2. Results and Discussion

We describe the results obtained in Sections 2.1–2.5 and discuss them in Sections 2.6.1–2.6.3.

### 2.1. Pre-Incubation with Nanomolar α-T for 3–18 h Protects PC12 Cells against H_2_O_2_-Induced Death; the Protective Effect of α-T Is Concentration-Dependent in the Nanomolar Range (1 nM < 10 nM < 100 nM) if Pre-Incubation of PC12 Cells with It Is Performed for 18 h

**Figure 1:**
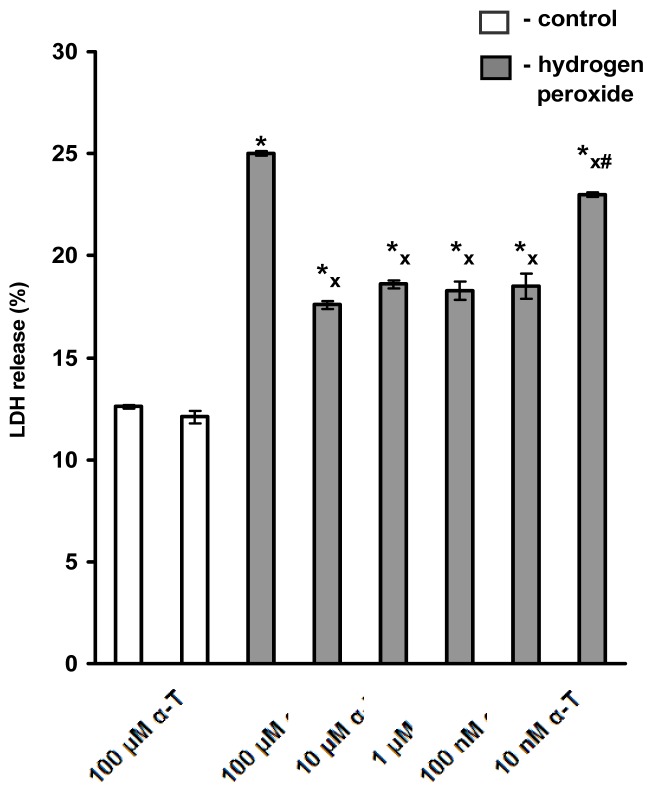
The figure shows that α-T at concentrations of 100 nM, 1 μM, 10 μM or 100 μM has a pronounced cytoprotective effect on the viability of PC12 cells if pre-incubation with α-T is performed for 18 h prior to exposure of the cells to 0.2 mM H_2_O_2_ for 24 h. No significant difference is revealed in the effect of α-T in these concentrations. The effect of 10 nM α-T is lower than the effect of all higher concentrations of this compound tested, but it is significant. In this figure, the results of one typical experiment (from five replicates) are presented as means ± SEM of 2–3 parallel determinations. The differences are significant by one-way ANOVA followed by Tukey’s multiple comparison test: * as compared to control values, *p* < 0.01; ^x^ as compared to the effect of H_2_O_2_ alone, *p* < 0.05; ^#^ as compared to the effect of α-T at all higher concentrations, *p* < 0.01.

If pre-incubation with α-T was performed for 18 h (*n* = 5) the rescue rates of 100 nM, 1 μM, 10 μM and 100 μM α-T against H_2_O_2_-induced cell death were found to be 48.3% ± 5.7%, 48.2% ± 7.8%, 47.1% ± 5.8% and 57.7% ± 4.2%, respectively (the difference between these values is not significant: *p* > 0.05 in all cases). Thus, the similar protection of PC12 cells against H_2_O_2_-induced death was achieved by long pre-incubation with α-T in the range from 100 nM to 100 μM. At a concentration of 10 nM, α-T still significantly inhibited the toxic effect of H_2_O_2_ by 29.6% ± 3.6% (*p* < 0.01), albeit to a lower extent than α-T at the higher concentrations tested (*p* < 0.02). The results of a typical experiment are shown in Figure 1.

**Figure 2:**
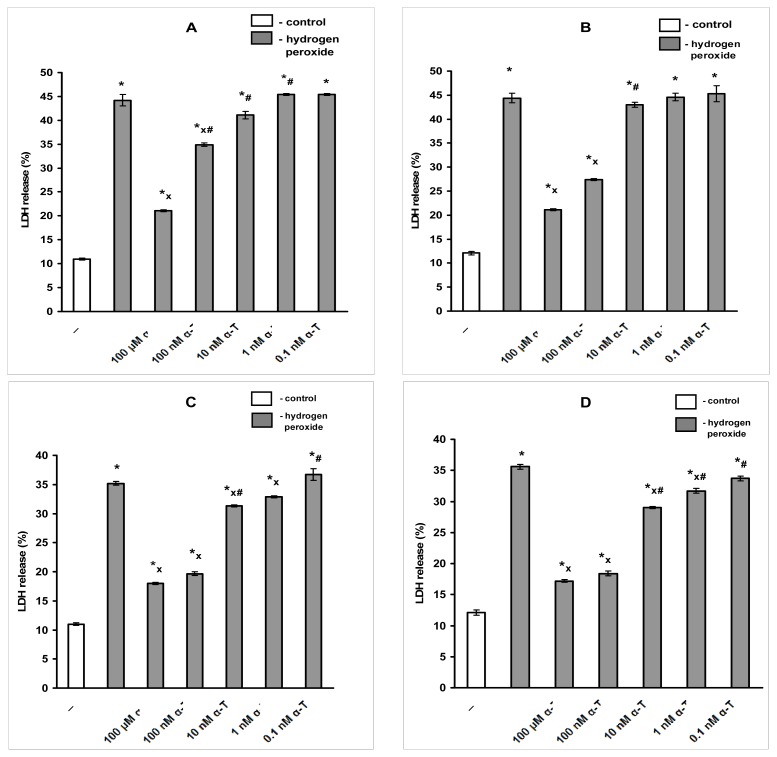
The figure provides evidence that the marked protective effect of 100 nM α-T is revealed when pre-incubation of PC12 cells with it is performed for 3–18 h, prior to the exposure to H_2_O_2_. In this figure, the results of one typical experiment (from four replicates) are presented as means ± SEM of 2–3 parallel determinations. PC12 cells were pre-incubated for 3 h (**A**); 6 h (**B**); 12 h (**C**) or 18 h (**D**) with α-T and then exposed to 0.2 mM H_2_O_2_ for 24 h. The differences are significant by one-way ANOVA followed by Tukey’s multiple comparison test: * as compared to control values, *p* < 0.01; ^x^ as compared to the effect of H_2_O_2_ alone, *p* < 0.05; ^#^ as compared to the effect of α-T at all higher concentrations, *p* < 0.05.

If pre-incubation is performed for 3 or 6 h, the protective effect of 100 nM α-T is lower than the effect of 100 μM α-T (Figure 2A,B). However, no difference in the protective activity of α-T for these two concentrations is observed if pre-incubation takes place for 12 or 18 h (Figure 2C,D). The data obtained in four experiments were expressed as rescue rates of α-T at various concentrations at various time of pre-incubation. These results are shown in Table 1.

The data of Table 1 and Figure 2D provide evidence that the protective effect of α-T is concentration-dependent in the range 1–100 nM (1 nM < 10 nM < 100 nM) if pre-incubation is performed for 18 h. The data presented in Table 1 (like the previously discussed data of Figure 2) show that the protective effect of 100 nM α-T is lower than the effect of 100 μM α-T if pre-incubation with it is performed for 3 or 6 h, but no difference is revealed in the protective effect of α-T in these two concentrations at longer pre-incubation (12 or 18 h).

**Figure 3:**
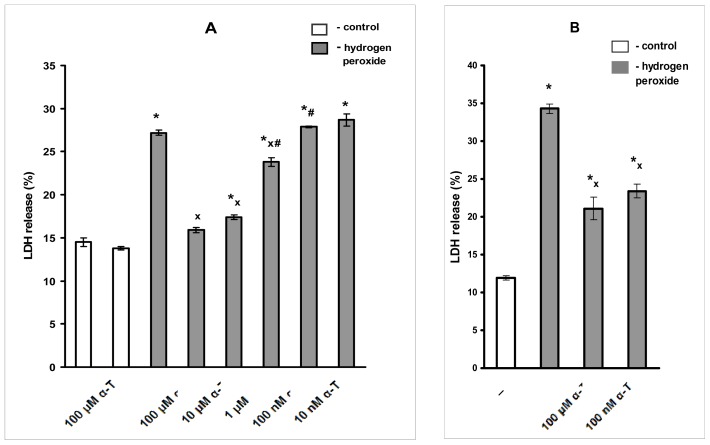
The figure shows the effects of pre-incubation with α-T for 0.5 and 18 h prior to PC12 cell exposure to 1 mM H_2_O_2_ for 3 h. (**A**) Pre-incubation with α-T at various concentrations for 0.5 h; (**B**) Pre-incubation with 100 nM and 100 μM α-T for 18 h. In this figure, the results of one typical experiment (from five experiments made) are presented as means ± SEM of 2–3 parallel determinations. The differences are significant by one-way ANOVA followed by Tukey’s multiple comparison test: * as compared to control values, *p* < 0.01; ^x^ as compared to the effect of H_2_O_2_ alone, *p* < 0.05; ^#^ as compared to the effect of α-T at all higher concentrations, *p* < 0.01. The data obtained provide evidence that pre-incubation with nanomolar α-T for 0.5 h prior to cell exposure to 1 mM H_2_O_2_ is not protective against H_2_O_2_-induced toxicity, while micromolar α-T is protective (**A**). However, if PC12 cells are pre-incubated with 100 nM or 100 μM α-T for 18 h and then exposed to 1 mM H_2_O_2_ (**B**) the protective effect of α-T in both concentrations is similar and significant (**B**).

We were interested to find out if α-T at nanomolar concentration was protective following short pre-incubation (0.5 h) and a short (3 h) exposure of 1 mM H_2_O_2_. It is to be emphasized that we used the cell exposure to 1 mM H_2_O_2_ for 3 h only in a very limited number of experiments presented in Figure 3. In all other figures and tables presented, the results of experiments were made using cell exposure to 0.2 mM H_2_O_2_ for 24 h.

These data show that even the toxicity of a high concentration of H_2_O_2_ (1 mM) may be overcome by long pre-incubation with nanomolar α-T (Figure 3B). The rescue rates of 100 nM and 100 μM α-T calculated using the data of all five experiments made were found to be 53.6% ± 8.6% and 59.1% ± 17.8%, respectively, if pre-incubation was performed for 18 h (*n* = 5).

### 2.2. Anti-Apoptotic Effect of α-T at Nanomolar and Micromolar Concentrations on PC12 Cells Exposed to Hydrogen Peroxide

**Figure 4:**
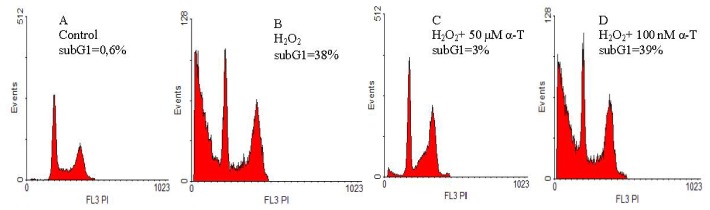
The figure shows effect of pre-incubation for 0.5 h with α-T prior to PC12 cell exposure to 0.2 mM H_2_O_2_ for 24 h on the relative number of PC12 cells in late apoptosis (% of the total cell number). The results of one typical experiment from four replicates are presented. The relative value of the subG1 hypodiploid peak (left on the histograms) corresponds to the relative number of PC12 cells in late apoptosis. In this figure: (**A**) control; (**B**) H_2_O_2_; (**C**) H_2_O_2_ + pre-incubation with 50 μM α-T; (**D**) H_2_O_2_ + pre-incubation with 100 nM α-T. In the control sample, the relative number of cells in late apoptosis (**A**) is less than 1%, but exposure to H_2_O_2_ markedly increases the number of cells in this peak (**B**). The pre-incubation of PC12 cells with 50 μM α-T for 0.5 h prior to their exposure to H_2_O_2_ decreases the hypodiploid peak from 38% (**B**) to 3% of total cell numbers (**C**), while pre-incubation with 100 nM is not effective, as it does not change the number of cells in this peak (**D**).

**Figure 5:**
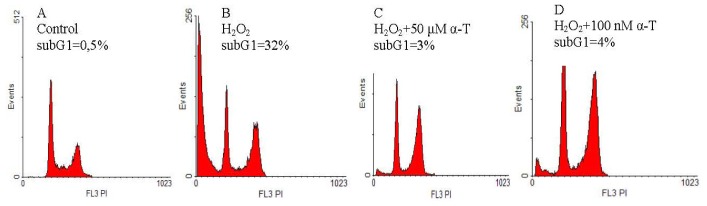
The figure shows the effect of pre-incubation for 18 h with α-T, prior to PC12 cell exposure to 0.2 mM H_2_O_2_ for 24 h on the relative number of PC12 cells in late apoptosis (% of the total cell number). The results of one typical experiment from 5 replicates are presented. The relative value of subG1 hypodiploid peak (left on the histograms) corresponds to the relative number of PC12 cells in late apoptosis. In the figure: (**A**) control; (**B**) H_2_O_2_; (**C**) H_2_O_2_ + pre-incubation with 50 μM α-T; (**D**) H_2_O_2_ + pre-incubation with 100 nM α-T. In the control sample, the relative number of cells in late apoptosis (**A**) is lower than 1%, but exposure to H_2_O_2_ increases this peak to 32% (**B**). However, pre-incubation with 50 μM and 100 nM α-T decreases the relative value of the subG1 hypodiploid peak from 32% (**B**) to 3% and 4% of the total cell number, respectively (**C**,**D**).

The anti-apoptotic effect of α-T was studied using cell staining with propidium iodide and flow cytometric analysis. The relative value of subG1 hypodiploid peak (left on the histograms) corresponds to the relative number of PC12 cells in late apoptosis. We compared the effect of short (0.5 h) and long (18 h) pre-incubation of PC12 cells with 100 nM and 50 μM α-T prior to their exposure to 0.2 mM hydrogen peroxide for 24 h (Figures 4 and 5, respectively).

It was found that 100 nM and 50 μM α-T possessed pronounced and similar anti-apoptotic activity on PC12 cells exposed to H_2_O_2_ if pre-incubation was performed for 18 h (Figure 5). However, α-T at nanomolar concentration was not effective if pre-incubation with it was performed for 0.5 h (Figure 4), while α-T in micromolar concentration had a pronounced anti-apoptotic effect under any conditions of the experiment. In each experiment, two parallel determinations were made; they produced the similar results.

The average percent of inhibition of the pro-apoptotic effect of H_2_O_2_ by α-T was calculated, taking into account the results of all experiments made. Pre-incubation of PC12 cells with 50 μM α-T for 0.5 h is highly effective, and decreases the number of PC12 cells in late apoptosis induced by H_2_O_2_ by 74.5% ± 8.2% (*p* < 0.01, *n* = 4). At the same time, pre-incubation with 100 nM α-T for 0.5 h does not produce any effect, as it inhibits the pro-apoptotic action of H_2_O_2_ by 9.2% ± 8.8% (*p* > 0.05, *n* = 4). When PC12 cells are pre-incubated with α-T for 18 h, α-T at concentrations 100 nM and 50 μM diminishes the pro-apoptotic effect of H_2_O_2_ by 63.8% ± 11.0% and 81.5% ± 9.0%, respectively (*p* < 0.01 in both cases, *n* = 5), the difference between the α-T effect at these two concentrations is not significant (*p* > 0.05).

### 2.3. Pre-Incubation with α-T at Nanomolar Concentrations for 18 h (But Not for 0.5 h) Protects Immature Cortical Neurons from H_2_O_2_-Induced Cell Death

**Figure 6:**
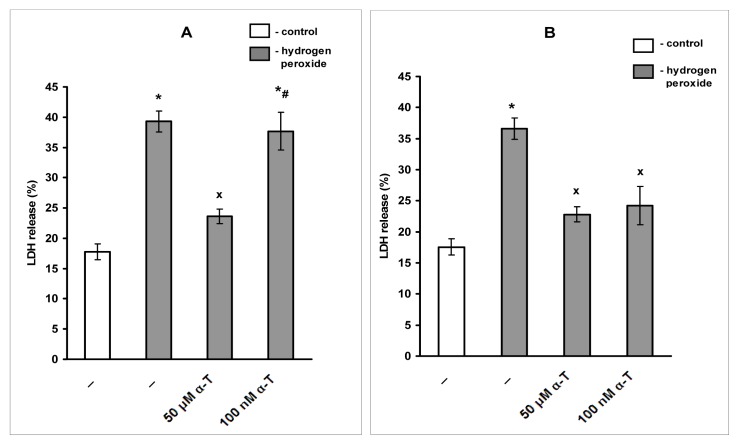
The figure shows the effect of pre-incubation of immature cortical neurons with nanomolar and micromolar α-T for 0.5 and 18 h on the viability of these cells exposed to hydrogen peroxide. The results of one typical experiment (*n* = 6) are presented as means ± SEM of 3–4 parallel determinations. The neurons were isolated from the brain cortex of an embryonic rat brain as described under Experimental procedure. After 6 days in culture (at the 7th day *in vitro*) immature cortical neurons were pre-incubated for 0.5 h (**A**) or 18 h (**B**) with α-T and then exposed to 0.2 mM H_2_O_2_ for 24 h. The differences are significant by one-way ANOVA followed by Tukey’s multiple comparison test: * as compared to control values, *p* < 0.01; ^x^ as compared to the effect of H_2_O_2_ alone, *p* < 0.01; ^#^ as compared to the effect of H_2_O_2_ and 50 μM α-T, *p* < 0.05.

The protective effect of pre-incubation of immature cortical neurons with 100 nM and 50 μM α-T for 18 h prior to their exposure to H_2_O_2_ was found to be pronounced and similar (Figure 6B). However, pre-incubation of cortical neurons for 0.5 h with 100 nM α-T is not effective, while pre-incubation for 0.5 h with 50 μM α-T increases the viability of immature neurons to a great extent (Figure 6A,B).

The rescue rates of α-T at concentrations 50 μM and 100 nM against H_2_O_2_-induced cell death of cortical neurons amounted in our study to 62.1% ± 9.8% and 59.0% ± 9.1%, respectively (*n* = 6), if pre-incubation with α-T was performed for 18 h. The difference between these values was not significant (*p* > 0.05). However, if cortical neurons were pre-incubated with α-T for 0.5 h, the rescue rates of 50 μM and 100 nM α-T constituted 54.2% ± 5.8% and 4.4% ± 4.6%, respectively. The difference between the effect of α-T at these two concentrations was significant (*p* < 0.001), but the effect of 100 nM α-T was absent (*p* > 0.05).

### 2.4. The Protective Effect of Pre-Incubation with α-T for 18 h against H_2_O_2_-Induced Toxicity in PC12 Cells Is Diminished or Abolished in the Presence of the Inhibitors of PKC, MEK 1/2 And PI 3-Kinase (GF109203X, SL327 and LY294002, Respectively)

We used the inhibitor of MEK 1/2 (SL327) that inhibits ERK 1/2 phosphorylation and activation, the inhibitor of PI 3-kinase (LY294002) that inhibits phosphorylation and activation of downstream Akt (protein kinase B), and the inhibitor of PKC (GF109203X). The data presented in Table 2 provide evidence that in the presence of PKC inhibitor (1 μM GF109203X), the ability of 100 nM and 100 μM α-T to rescue PC12 cells from H_2_O_2_ -induced cell death is diminished and becomes insignificant. In the presence of 50 μM LY294002 or in the presence of 10 μM SL327, the protective effect of 100 nM and 100 μM α-T is significantly lower (*p* < 0.05) than in the absence of these inhibitors (Table 2). However, 10 μM LY294002 is ineffective, as this concentration does not appear to be sufficient to cause the total inhibition of PI 3-kinase activity [19]. One microliter SL327 or 0.1 μM GF109203X are also ineffective (data not shown).

### 2.5. While α-T Decreases the Time of Maximal Activation of ERK 1/2 and Akt in PC12 Cells Initiated by H_2_O_2_, the Effect of 100 nM and 100 μM α-T Is Similar

One of the main aims of our study is to reveal how α-T modulates the activity of ERK 1/2 and Akt under conditions of oxidative stress induced by H_2_O_2_ by using immunoblotting. The results of such study will be given in the present section. We suggest that modulation of these protein kinase activities by α-T under conditions of oxidative stress makes a contribution to its protective effect against H_2_O_2_-induced toxicity.

We studied the effect of α-T on basal pERK 1/2 and pAkt levels in PC12 cells as well. It was difficult to make studies of the basal pERK 1/2 level as it was found to be very low in PC12 cells and diminished even more as a result of cell exposure to α-T.

**Figure 7:**
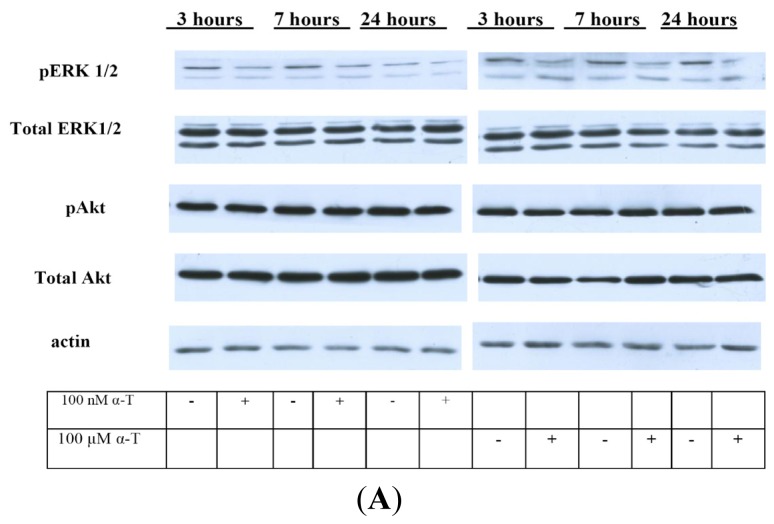

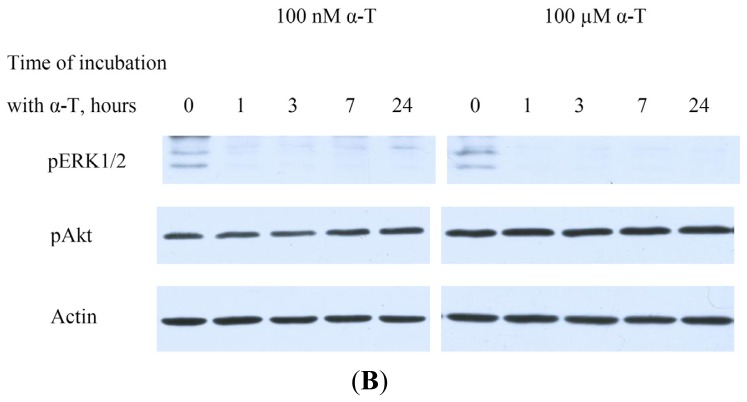
The figure shows that the level of pERK 1/2 decreases 1, 3, 7 and 24 h after the exposure of PC12 cells to 100 nM and 100 μM α-T, but there is no change in the total level of ERK 1/2 and Akt and in the level of pAkt in these cells as a result of such exposure. The data of one typical experiment from 3 experiments made are presented. (**A**) the data were obtained using ECL PLUS (Amersham) to enhance the chemiluminescent signal; (**B**) SuperSignal West Pico Chemiluminescent Substrate (Pierce, Thermo Scientific) was used as an enhancement solution.

If we used SuperSignal West Pico Chemiluminescent Substrate (Pierce, Thermo Scientific) as an enhancement solution (Figure 7B), the basal pERK activity was revealed on the blots only in controls, but after PC12 cell exposure to 100 nM and 100 μM α-T for 1, 3, 7 and 24 h, it was practically absent.

Thus, pERK 1/2 level was found to be low in PC12 cells, while the cell exposure to α-T was found to diminish even more the basal pERK 1/2 level at various times of cell exposure to α-T. No difference in the basal level of ERK 1/2, Akt or pAkt was revealed in PC12 cells as a result of cell exposure to 100 nM or 100 μM α-T for various time intervals.

It was of importance to study the effect of protein kinase inhibitors on the expression and activity of ERK 1/2 and Akt in PC12 cells exposed to hydrogen peroxide.

**Figure 8:**
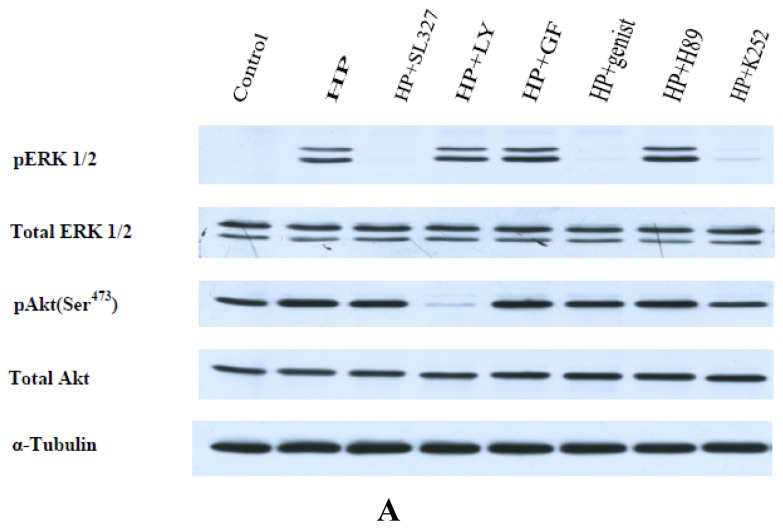

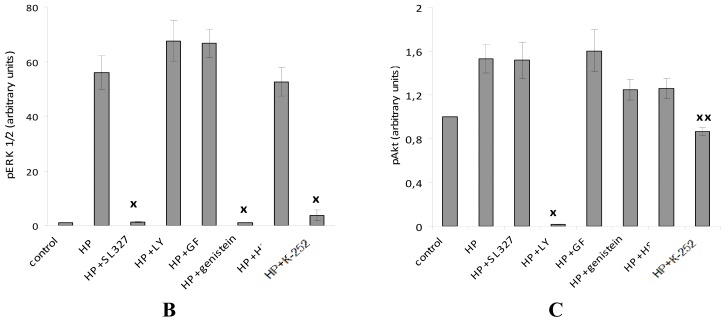
The figure shows the effect of pre-incubation for 30 min with protein kinase inhibitors before cell exposure to 0.3 mM H_2_O_2_ for 20 min on ERK 1/2, Akt, pERK 1/2 and pAkt level in PC12 cells. In this figure, HP is hydrogen peroxide. (**A**) Results of immunoblotting studies; (**B**) Effect of protein kinase inhibitors on pERK 1/2 level in PC12 cells; (**C**) Effect of protein kinase inhibitors on pAkt level in PC12 cells. In (**B**,**C**): ^x,xx^ the difference with the values obtained after exposure to H_2_O_2_ alone is significant; ^x^ *p* < 0.01; ^xx^ *p* < 0.05. In the figure, LY is 50 μM LY294002, GF is 1 μM GF109203X, genist is 100 μM genistein, K252 is 1 μM K252a, 10 μM SL327 and 10 μM H89 were used.

**Figure 9:**
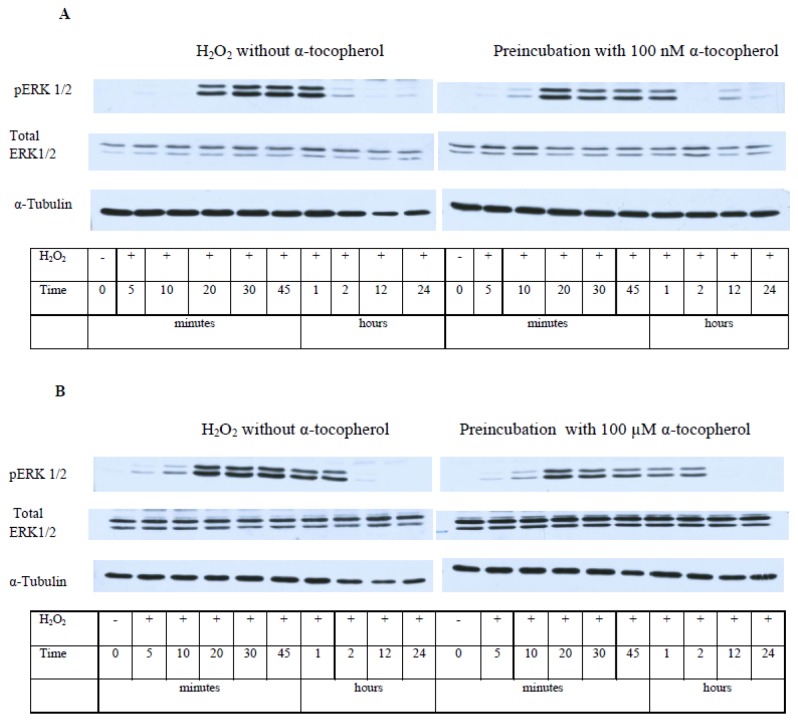

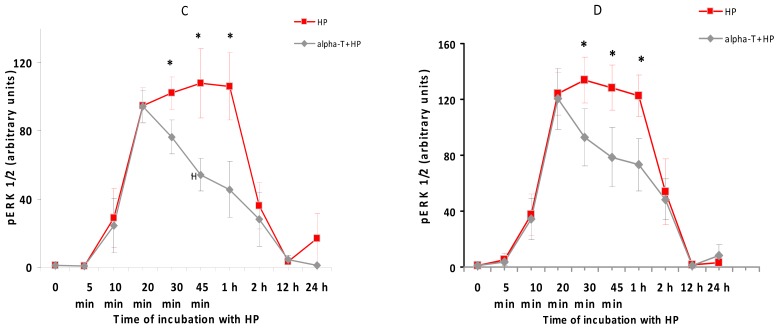
The figure shows the effect of hydrogen peroxide and pre-incubation with α-T on the activity (pERK 1/2 level) and expression of ERK 1/2 in PC12 cells. PC12 cells were pre-incubated with 100 nM and 100 μM α-T (or without it) for 18 h and then exposed to 0.3 mM H_2_O_2_. The results of immunoblotting obtained in one typical experiment (from five experiments made) are shown in (**A**,**B**), (**A**) 100 nM α-T; (**B**) 100 μM α-T. The results of five experiments are shown in (**C**,**D**) as means ± SEM; (**C**) 100 nM α-T; (**D**) 100 μM α-T. Red lines with square data points show effect of H_2_O_2_ alone, black lines with diamond data points show the effect of H_2_O_2_ after pre-incubation with α-T. In (**C**,**D**): HP is an abbreviation for hydrogen peroxide. alpha-T is an abbreviation for α-tocopherol * the differences are significant according to Students’ paired *t* test, as compared to the level of pERK 1/2 in the PC12 cells exposed to α-T and H_2_O_2_, *p* < 0.05.

**Figure 10:**
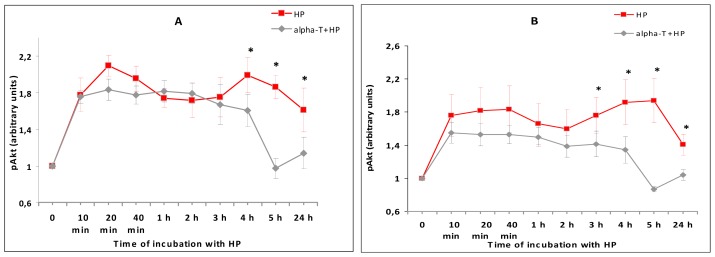
The figure shows effect of pre-incubation of PC12 cells with 100 nM and 100 μM α-T for 18 h prior to exposure of cells to 0.3 mM H_2_O_2_ on the activity of Akt (pAkt level). The results of five experiments made are shown as means ± SEM. (**A**) Pre-incubation with 100 nM α-T; (**B**) Pre-incubation with 100 μM α-T. Red lines with square data points show effect of H_2_O_2_ alone, black lines with diamond data points show effect of H_2_O_2_ after pre-incubation with α-T. On (**A**,**B**): HP = hydrogen peroxide; alpha-T = α-tocopherol. * The differences are significant according to Students’ paired *t* test as compared to the level of pAkt in the PC12 cells exposed to α-T and H_2_O_2_, *p* < 0.05.

As Figure 8 shows, in the presence of 10 μM SL327, pERK 1/2 is not revealed in PC12 cells, while in the presence of 50 μM LY294002, pAkt is absent in these cells. The effect of other inhibitors used will be described later (after Figures 9 and 10).

The data presented in Figure 9A,B show that a well-pronounced and extended increase of pERK 1/2 levels in PC12 cells after their exposure to H_2_O_2_ is observed for 20–60 min, but no changes in total ERK 1/2 levels is revealed. These data provide evidence that ERK 1/2 is activated in PC12 cells by H_2_O_2_, but the level of its expression is not changed. The maximal level of pERK 1/2 is achieved in 20 min after PC12 cell exposure to hydrogen peroxide and then it is maintained at a high level for a rather long time, forming a plateau (Figure 9C,D). α-T does not change the initial increase in pERK 1/2 levels up to 20 min after cell exposure to H_2_O_2_. However, α-T at nanomolar and micromolar concentrations causes a significant and pronounced decrease of the pERK 1/2 level 30, 45 and 60 min after the exposure of PC12 cells to H_2_O_2_ (Figure 9A–D). In cells exposed both to α-T and H_2_O_2_ (as compared to cells exposed to H2O2 alone), a peak instead of a plateau is seen on the histograms (Figure 9C,D).

The studies of the effects of protein kinase inhibitors on pERK 1/2 levels were made during exposure to H_2_O_2_ for 20 min, as maximal activation of ERK 1/2 by H_2_O_2_ (maximal pERK 1/2 level) was already achieved after the same cell exposure time to H_2_O_2_. As Figure 8 shows, the basal pERK 1/2 level is very low, but H_2_O_2_ increases it to a great extent. As it was already mentioned, in the presence of MEK 1/2 inhibitor SL327, pERK l/2 was not practically revealed in cells exposed to H_2_O_2_, but total ERK 1/2 level was not changed. The inhibitors of PKC (1 μM GF109203X), of protein kinase A (1 μM H89) and of PI 3-kinase (50 μM LY LY294002) had no effect either on total ERK 1/2 or on pERK 1/2 level in PC12 cells exposed to hydrogen peroxide. It is of interest that the inhibitor of Trk receptor tyrosine kinase (1 μM K252a) was found to decrease significantly pERK 1/2 levels in PC12 cells exposed to H_2_O_2_. These data suggest that at early stages of ERK activation by H_2_O_2_, it may be activated downstream of Trk receptor tyrosine kinase. Trk receptors are neurotrophin receptors and activation of Trk receptor tyrosine kinase is usually protective.

The exposure of PC12 cells to H_2_O_2_ was found to result in a pronounced and prolonged increase of Akt activity (increase in pAkt levels) in the cells (Figure 10A,B), while the total Akt level did not change (data not shown). Pre-incubation of PC12 cells for 18 h with 100 μM or 100 nM α-T did not change pAkt level for the first 2–3 h after cell exposure to H_2_O_2_, but pAkt levels were significantly lower in cells exposed to α-T and H_2_O_2_ than in cell exposed to H_2_O_2_ alone 4, 5 and 24 h after the cell exposure to H_2_O_2_ (Figure 10A,B).

The studies of effects of protein kinase inhibitors on pAkt level were made using exposure to H_2_O_2_ for 20 min because maximal activation of Akt by H_2_O_2_ (maximal pAkt level) was achieved after the same period of cell exposure to H_2_O_2_ (Figure 10). The basal level of Akt activity is pronounced; exposure to H_2_O_2_ significantly increases it (Figure 8). As previously mentioned, in the presence of PI 3-kinase inhibitor 50 μM LY294002, pAkt was not revealed, but total Akt level was not changed. Neither did the inhibitors of PKC (1 μM GF109203X), of protein kinase A (10 μM H89) or of MEK 1/2 (10 μM SL327) have an effect on Akt expression, or on pAkt levels in PC12 cells exposed to H_2_O_2_. It is to be noted that the inhibitor of Trk receptor tyrosine kinase (1 μM K252a) was found to decrease significantly pAkt level in PC12 cells exposed to H_2_O_2_.

Thus, our immunoblotting data show that H_2_O_2_ activates both ERK 1/2 and Akt (increases pERK 1/2 and pAkt levels), but does not change the expression of these protein kinases. The pre-incubation with α-T prior to PC12 cell exposure to H_2_O_2_ diminishes the time of maximal activation of these protein kinases induced by H_2_O_2_.

### 2.6. Discussion

#### 2.6.1. α-T and the Function of the Central Nervous System

The central nervous system is very sensitive to vitamin E or α-T deficiency. The lack of α-T in various organs of the human body caused by abnormal α-T transfer protein synthesis (as a result of gene mutations) leads to a disease with predominantly neurological symptoms called “ataxia with vitamin E deficiency”. Additional vitamin E administration appears to be helpful in such cases [20]. However, the clinical trials of vitamin E administration to patients with Alzheimer’s or other neurodegenerative diseases did not provide for favorable results; therefore, it was supposed that, probably, it is “time to stop feeding vitamin E to dementia patients” [21]. Additionally, the effects of vitamin E administration to patients with various diseases were analyzed using the results of all published randomized clinical trials which included more than 100,000 observations. It was found that vitamin E supplementation of the diet at high doses significantly increased the all-causes mortality in adult patients as well as for other people in the risk groups [22,23]. It should be indicated that α-T concentrations in the extracellular spaces of organs which have a blood-organ barrier (like the blood-brain barrier) may differ from its extracellular concentration in organs which do not have such barrier.

#### 2.6.2. α-T at Nanomolar Concentration Protects PC12 Neuronal Cell Line and Cultured Immature Cortical Neurons from H_2_O_2_-Induced Cell Death If Pre-Incubation Is Long, Nanomolar α-T Has Anti-Apoptotic Effect on PC12 Cells

In our studies, α-T at nanomolar concentration was found to protect PC12 cells from H_2_O_2_-induced cell death if pre-incubation with it was performed for 3–18 h. Using PC12 cells, we showed for the first time that the ability of α-T to decrease the oxidative stress-induced death of PC12 cells depended on its concentration in the nanomolar range (1 nM < 10 nM < 100 nM) if pre-incubation time was 18 h. At such time of pre-incubation, the maximal protective effect was achieved by 100 nM α-T: the rescue rates of 100 nM, 1 μM, 10 μM and 100 μM α-T were not different. We do not know of any other study showing that the protective effect of α-T on cells may be concentration-dependent in the nanomolar range.

Numakawa and co-authors [17] were the first to show that nanomolar α-T protected immature cortical neurons from H_2_O_2_ toxicity if pre-incubation was performed for a long time. Our results confirm these data. No difference was revealed in rescue rates of 100 nM α-T or micromolar α-T using different methods to assess the cell viability—LDH method in our study and MTT method in the above-mentioned study [17].

Another evidence of protection by pre-incubation with nanomolar α-T was recently obtained studying PC12 cell death induced by eleostearic acid, which was accompanied by (PARP)-1 independent apoptosis-inducing factor release and activation of reactive oxygen species production in the cells [18]. In this case, PC12 cell death could be blocked by the presence of 23 nM α-T in the medium [18].

Neither primary cultures of immature neurons, nor neuronal cell lines, appear to be the ideal models to study the effect of toxins and protectors on the survival of nerve cells *in vivo*. Thus, nerve cells in primary cultures differ to a great extent one from another and from adult brain neurons, as far as the presence of various receptors is concerned. It is very difficult, if impossible, to avoid the presence of large numbers of glial cells if cortical neurons are given time to become more “mature” *in vitro*. So the conclusions seem more reliable if similar data are obtained using various cell types. The protective effect of nanomolar α-T against H_2_O_2_-induced cell death was shown using both PC12 neuronal cell line and immature cortical neurons. It suggests that α-T at physiological concentrations characteristic for the brain extracellular space has a protective effect on brain neurons.

We have found that 100 nM and 100 μM α-T have similar protective effects on PC12 cells under conditions of oxidative stress if pre-incubation with it is performed for 18 h. The radical scavenging activity of micromolar and nanomolar α-T greatly differs. However, nanomolar α-T may accumulate in cell membranes during the long pre-incubation of cells. We have failed to find in the literature data showing if long incubation of PC12 cells with nanomolar α-T leads to an increase of its content in the membranes of these cells. Such data would show if the protective effect of long pre-incubation of PC12 cells with nanomolar α-T depends (or does not depend) on its accumulation in cell membranes.

Apoptosis or necrosis may cause cell death induced by H_2_O_2_. Apoptotic cells in the absence of phagocytosis are known to proceed to secondary necrosis, such cells have many features of primary necrotic cells [24,25]. Using flow cytometry, we revealed a high percentage of PC12 cells in late apoptosis after their exposure to 0.2 mM H_2_O_2_ for 24 h, pre-incubation for 18 h with 100 nM, and 50 μM α-T had a pronounced and similar anti-apoptotic effect (Figure 5). However, if PC12 cells were pre-incubated with α-T for 0.5 h, only micromolar α-T had an anti-apoptotic activity, while nanomolar α-T was ineffective (Figure 4). Apoptotic cell death appears to predominate if PC12 cells are exposed to 0.1–0.2 mM H_2_O_2_ [26]. Thus, it is suggested that secondary necrosis might have made a great contribution to PC12 cell death in our experiments, while the increase of cell viability by long pre-incubation with nanomolar and micromolar α-T may be explained to a large extent by its anti-apoptotic activity.

The protective effect of α-T depends to a great extent on the time of pre-incubation prior to cell exposure to toxins. In contrast, nanomolar α-tocotrienol is protective if added to the medium 5 min before or even after cell exposure to glutamate [14–16]. However, pre-incubation for 0.5 h is not sufficient to reveal protection by nanomolar α-T in cortical neurons or to reveal its anti-apoptotic effect in PC12 cells ([17] and the present work, respectively). The long pre-treatment (16 h or more) of hippocampal neurons with 1–2.5 μM α-T prior to induction of oxidative stress by Fe^2+^ is needed to provide a long-lasting protection. The authors indicate that this is in contrast to the transient effect of 10 μM α-T, based on its radical scavenging activity [1,7].

#### 2.6.3. The Modulation of ERK 1/2, Akt and PKC Activity by Nanomolar and Micromolar α-T and Its Contribution to α-T Protective Effect in PC12 Neuronal Cell Line

We studied the possible contribution of modulation of ERK 1/2, Akt and PKC activity to the protective effect of α-T in PC12 cells. However, it is to be noted that the protective effect of α-T may also depend on modulation of other signaling pathways.

The prolonged activation of ERK 1/2 in PC12 cells by H_2_O_2_ revealed by us is in agreement with data showing the activation of this enzyme by reactive oxygen species [27–29]. Pre-incubation with nanomolar and micromolar α-T for 18 h did not change the initial activation of ERK 1/2 during the first 20 min after PC12 cell exposure to H_2_O_2_, but it markedly decreased the time of maximal activation of ERK 1/2 by H_2_O_2_. It is of interest that the similar effect of carnosine on ERK 1/2 activity was recently described, carnosine was shown to protect cerebellar granule cells against oxidative stress and to decrease the time of ERK 1/2 activation [29]. Such an effect of α-T in our studies may result from activation of protein phosphatases, especially 2A [3,4,30]. Both α-T and its derivative which does not have radical scavenging activity were shown to inhibit ERK 1/2 activity [31]. 100 nM and 100 μM α-T had a similar effect (Figure 9) on ERK 1/2 activity (if pre-incubation with α-T was performed for 18 h); such data suggest a non-antioxidant mechanism of an α-T inhibitory effect.

Activation of ERK 1/2 was at first considered to increase cell viability. However, the excessive activation of ERK 1/2 was shown to lead to the death of immature cortical neurons and cells of a neuroblastoma line [32,33]. Thus, activation of the Ras/MEK/ERK 1/2 pathway leads to mitochondrial dysfunction and death of cultured cortical neurons exposed to toxic zinc concentrations [32]. Oxidative stress induced by glutathione depletion leads to sustained activation of ERK 1/2 and death of immature cortical neurons and cells of the HT22 neuroblastoma line [33].

In our studies, the inhibitor of MEK 1/2 SL327 prevents the ERK 1/2 activation by H_2_O_2_ in PC12 cells under conditions of our experiments (Figure 8). However, it does not change the viability of PC12 cells exposed to H_2_O_2_. An increase of LDH release caused by cell exposure to H_2_O_2_ and SL327 constituted 102% ± 7.6% of the increase of LDH release in PC12 cells exposed to H_2_O_2_ alone (taken for 100%), the difference being not significant. It is of interest that in another work [17] MEK 1/2 inhibitor (UO126) was also found not to have significant effect on cell viability under conditions of oxidative stress; this inhibitor did not change the viability of cultured immature cortical neurons exposed to hydrogen peroxide [17]. At the same time, the protective effect of α-T on PC12 cells under conditions of oxidative stress was found to be diminished in the presence of SL327. It is of interest that activation of ERK 1/2 by H_2_O_2_ during the first 20 min of PC12 cell exposure to H_2_O_2_ could be prevented not only by SL327, but also by the inhibitor of Trk receptor tyrosine kinase K252a (Figure 8). We suggest that there is a difference in the effect of early and late stage ERK 1/2 activation by H_2_O_2_ on PC12 cell viability. The initial stages of ERK 1/2 activation appear to be protective, as usually is the activation of Trk receptor tyrosine kinase and of protein kinases activated downstream, including ERK 1/2. However, if activation of ERK 1/2 is long, the late stages of ERK activation may decrease cell viability. α-T inhibits the late stages of ERK 1/2 activation by H_2_O_2_ (Figure 9) and increases the viability of PC12 cells. However, the total inhibition of ERK 1/2 by SL327 (Figure 8) is not protective. If the effect of α-T is studied in the presence of SL327, its protective effect is diminished as both the initial (protective) and the late stages of ERK 1/2 activation by H_2_O_2_ are inhibited. According to the data of Luo and co-authors (who studied the effect of glutamate-induced oxidative toxicity, mediated by glutathione depletion on the viability of HT22 mouse hippocampal cell line) ERK 1/2 activation may be protective in early phases of its activation, but it may contribute to toxicity during later phases of oxidative stress [27].

It is of interest to compare our data with the data obtained by Numakawa and co-authors studying α-T effects on cultured immature cortical neurons [17]. They found that α-T increased the basal activity of ERK 1/2 in these cells, while in the presence of the inhibitor of this protein, kinase protective effect of α-T was not revealed. It may be the case as the activation of ERK 1/2 by neurotrophins and flavanones appears to contribute to their protective effect in neuronal cell lines or cultured neurons [34,35]. However, the simple explanation that ERK activation increases the cell survival of cortical neurons, while its inhibition leads to their death, does not work in this model [17], as in our case. As hydrogen peroxide is known to activate ERK 1/2 [27–29] the inhibition of ERK 1/2 activity by MEK 1/2 inhibitor (UO126) could be suggested (according to such an explanation) to result in a marked decrease of the viability of the cortical neurons. However, the experimental data obtained by Numakawa and co-authors show that MEK 1/2 inhibitor does not diminish the viability of cortical neurons exposed to H_2_O_2_. Figure 5A [17] clearly shows that the viability of immature cortical neurons exposed to H_2_O_2_ or to H_2_O_2_ in the presence of UO126 is quite similar (the inhibitor did not change the viability of control cortical neurons as well).

In PC12 cells, α-T did not increase the basal pERK 1/2 level, on the contrary, it diminished it. As the data showing how α-T modulates ERK 1/2 and other protein kinase activities of immature cortical neurons under conditions of oxidative stress have not been obtained yet, it is not clear if activation of basal ERK 1/2 activity in cortical neurons during pre-incubation with α-T is followed, or not, by its inhibition on late stages of ERK 1/2 activation by H_2_O_2_.

The sustained activation of ERK 1/2 in brain or spinal cord cells subjected to ischemia and reperfusion leads to neuronal death, while administration of ERK 1/2 inhibitors markedly increases the survival of brain neurons [36–38]. Further studies will provide evidence of how α-T modulates the activity of ERK 1/2 in brain neurons under conditions of oxidative stress. Our data suggest that the decrease of time of ERK 1/2 maximal activation in neurons by α-T under conditions of oxidative stress may be protective.

H_2_O_2_ causes long-lasting activation of Akt in PC12 cells. In the presence of PI 3-kinase inhibitor LY294002, this activation is abolished (Figure 8). The presence of this inhibitor in the medium increases the death of PC12 cells exposed to H_2_O_2_. Thus, the LDH release from PC12 cells as a result of their exposure to LY294002 and H_2_O_2_ constitutes 139.7% ± 5.7% of LDH release from the cells exposed to H_2_O_2_ alone (taken for 100%), the difference is significant (*p* < 0.05). According to our data, α-T inhibits Akt only during late stages of its prolonged activation by H_2_O_2_ (Figure 9). If α-T inhibited the effect of Akt both at the early and at the late stages of its activation by H_2_O_2_, its effect would be toxic, like the effect of LY294002. Our data on the inhibitory effect of α-T on Akt activity are in agreement with the data of other authors [3,4]. Such an effect may also mediate the protective effect of long pre-incubation with nanomolar and micromolar α-T against H_2_O_2_-induced PC12 cell death, as the rescue rates of α-T decrease in the presence of a PI 3-kinase inhibitor.

There are numerous data showing that α-T inhibits PKC activity [2,5,39–41]. Thus, α-T inhibits PKCδ activity in the hippocampus dentate gyrus neurons *in vivo* [2]. Maternal dietary load of α-T leads to a marked decrease of PKC phosphorylation in rat offspring [39]. α-T inhibits activity of PKC from rabbit hearts in a broad concentration range, 100 nM–100 μM α-T is a potent inhibitor of PKC [40]. α-T, but not β-tocopherol, inhibits PKC in endothelial cells [41]. It is difficult to describe what effects the inhibition of PKC activity has on the viability of the cells, as there are numerous forms of these enzymes, and they may be localized in various cell compartments. The literature data provide evidence that the inhibition of PKC may lead both to the increase of cell viability and to cell death.

In our experiments in the presence of PKC inhibitor, the protective effect of long pre-incubation with nanomolar and micromolar α-T against H_2_O_2_-induced toxicity was practically abolished. It seems that the protective effect of α-T is not due to inhibition of all forms of protein kinase C available in PC12 cells. Thus, in our experiments, the PKC inhibitor GF109203X did not increase the viability of PC12 cells exposed to H_2_O_2_. The LDH release from PC12 cells as a result of their exposure to GF109203X and H_2_O_2_ constitutes 102.3% ± 1.8% of LDH release from these cells exposed to H_2_O_2_ alone (taken for 100%), the difference is not significant. If the cells are exposed to H_2_O_2_, α-T and GF109203X, the LDH release does not significantly differ from the release induced by H_2_O_2_ alone as well. It may be suggested that activation of some forms of PKC or activation of PKC in some compartments of the cells (for example downstream of neurotrophin receptors) may be protective in PC12 cells. Thus, for example, activation of PKC downstream of TrkB receptor tyrosine kinase was shown to increase survival of cerebellar granule cells [42]. There are other works showing that activation of certain forms of PKC in neurons and other cells may protect them from damage [43,44]. However, it is suggested that α-T may inhibit such forms of PKC (or PKC in such compartments of PC12 cells) whose activation leads to loss of cell viability. Such a suggestion may explain why the viability of PC12 cells does not increase in the presence of PKC inhibitor GF109203X, but does increase in the presence of α-T. There are numerous data showing an increase in cell viability as a result of PKC inhibition. Thus, for example, inhibition of this enzyme prevents the PKC-dependent mitochondrial translocation of pro-apoptotic protein Bax induced in cells of ventrolateral medulla by bacterial lipopolysaccharide [45]. PKCδ inhibition was shown to block effectively the activation of caspase-9 and caspase-3 during proteasome dysfunction of dopaminergic neuronal cells [46]. The protective effect of PKC inhibition by α-T may be a result of prevention of mitochondrial dysfunction and caspase activation in PC12 cells. It seems of interest to reveal if inhibition of PKC and ERK 1/2 activity by α-T in PC12 cells exposed to H_2_O_2_ may result in changes of expression and/or translocation to mitochondria of anti- and pro-apoptotic proteins.

## 3. Experimental Procedure

### 3.1. Materials

Hydrogen peroxide, pyruvate, NADH, cytosine-arabinoside, α-T and propidium iodide were obtained from Sigma (USA), penicillin and streptomycin were from Serva (Germany), K-252a, SL327, LY294002 and GF109203X were from Calbiochem (USA). The incubation media, Dulbecco’s Modified Eagle Medium (DMEM) with L-glutamine, horse blood serum and fetal calf serum were purchased from the Biolot Company (Russia). The antibodies used for immunoblotting are listed in Immunoblotting section.

### 3.2. PC12 Cells in Culture

The experiments were performed on PC12 cells (ATCC) in CO_2_ incubators in an atmosphere containing 5% CO_2_ at 37 °C. DMEM containing 10% fetal calf serum, 5% horse blood serum, 50 U/mL of penicillin and 50 μg/mL of streptomycin was used as the complete growth medium and it was changed every 2–3 days. PC12 cells were seeded onto 24-well plates at a density of 2 × 10^5^ cells per well to determine the cell viability. The experiments started 24 h after the transfer of the cells to the plates, and were performed in the complete growth medium. PC12 cells were pre-incubated with α-T for 3, 6, 12, or 18 h prior to cell exposure to 0.2 mM H_2_O_2_ for 24 h. After pre-incubation with α-T for 0.5 h (Figure 3A), and in some experiments (Figure 3B) for 18 h, PC12 cells were exposed to 1 mM H_2_O_2_ for 3 h. In some experiments, PC12 cells were pre-incubated in the presence of protein kinase inhibitors (SL327, LY294002, GF109203X) for 0.5 h, before the exposure of the cells to α-T.

### 3.3. Assessment of Cell Viability Using the Lactate Dehydrogenase (LDH) Method

The viability of PC12 cells and cortical neurons was assessed by measuring the activity of LDH released from the damaged cells after their exposure to H_2_O_2_. The samples were centrifuged before the aliquots of supernatant were taken. The activity of LDH was determined in the samples by measuring the decrease in NADH levels. This reaction was performed in medium with the following composition: 80 mM tris-HCl (pH = 7.2), 200 mM NaCl, 1.6 mM pyruvate, 0.2 mM NADH. The decrease in optical density of the samples at 340 nm was registered at approximately 5–6 min as previously described [47], using the M40 spectrophotometer (Karl-Zeiss, Germany). The lysis of the cells was performed using 1% Triton X-100 at room temperature in order to determine total LDH activity in the samples. The percent of LDH activity in the incubation medium was estimated in comparison to the total LDH activity. The absence of viable cells corresponds to 100% of LDH activity in the incubation medium.

The difference in the amount of LDH released from the cells exposed to H_2_O_2_ in the absence and in the presence of α-T was determined. The ratio of this difference to the increase of LDH released from cells to the medium in the presence of H_2_O_2_ alone (taken for 100%) corresponds to the rescue rates of α-T against H_2_O_2_ toxicity (*i.e*., to the percent of inhibition of H_2_O_2_ toxic action by α-T). The formula is

$$\frac{\text{LDH release in }{\text{H}}_{2}{\text{O}}_{2}-\text{LDH release in }{\text{H}}_{2}{\text{O}}_{2}\;\text{and }\alpha -\text{T}}{\text{LDH release in }{\text{H}}_{2}{\text{O}}_{2}-\text{LDH release in control}}\times 100$$

### 3.4. Isolation of Embryonic Rat Cortical Neurons

Primary cultures of immature cortical neurons were prepared from embryonic day 16 Wistar rat fetuses by the modified method of Dichter [48], as previously described [49]. Cells were seeded on poly-d-lysine coated 24-well plates at a density of 3 × 10^5^ cells per well. After 24 h, cytosine-arabinoside (3 μM) was added to the culture for 24 h in order to minimize glial growth. Culture medium was replaced every 3 days. Treatments were performed on the 7th day *in vitro*. Pre-incubation of neurons with α-T was performed for 0.5 or 18 h prior to the exposure of cells to 0.2 mM H_2_O_2_ for 24 h.

### 3.5. Flow Cytometric Analysis

After exposure to α-T and H_2_O_2_, PC12 cells were collected by centrifugation, rinsed in PBS, fixed with ice cold 70% ethanol and stored at −20 °C for 24 h–48 h. The cells were then incubated with a DNA extraction buffer as previously described [50], and washed with PBS. The cell pellets were resuspended in a DNA staining reagent (200 μg of RNase and 50 μg of propidium iodide in 1 mL PBS was used per 0.5–1 × 10^6^ cells) and incubated in the dark at 4 °C for 30 min. The amount of cells in hypodiploid (subG1) peaks and cell cycle distributions at different phases were analyzed using an EPICS XL flow cytometer (Beckman-Coulter, USA) and the WinMDI 2.1.4 software package. Between 40,000 and 80,000 cells were taken for the analysis of each sample. The relative content of DNA in the peaks was measured as the red propidium iodide fluorescence. The percent of cells in the subG1 peak corresponded to the relative number of cells undergoing late apoptosis in the sample.

### 3.6. Immunoblotting

The phosphorylated and non-phosphorylated forms of ERK1/2 and Akt were determined using Western blot analysis. After incubation with α-T and H_2_O_2_, PC12 cells were washed twice with ice-cold PBS and harvested in 60 μL of lysis buffer: 50 mM Tris pH 8.0, 150 mM NaCl, 1% Triton X-100, 5 mM EDTA, 10 mM β-glycerophosphate Na, 10 mM NaF, 1 mM Na_3_VO_4_, 1 mM phenyl methyl sulfonyl fluoride (PMSF), protease inhibitor cocktail (Roche). Cells were kept on ice for 1 h to complete lysis. Protein concentrations of the cell lysates were estimated in triplicate using the Lowry method with Folin & Ciocaltteu’s Phenol reagent and bovine serum as a standard. Equivalent amounts (35–40 μg) of protein-containing lysates were loaded into each lane and electrophoresed in 10% sodium dodecyl sulfate-polyacrylamide gel, followed by transfer to Hybond-P PVDF membranes (Amersham, GE Healthcare). The non-specific binding sites of the membranes were blocked with PBS containing 5% (*w*/*v*) skimmed milk and 0.1% Tween 20. The blots were then probed overnight with antibodies for pERK1 (pThr202/pTyr204) and pERK2 (pThr185/pTyr187) (1:2000, Sigma), pAkt (Ser473) (1:1000, Cell Signaling), followed by three washes with 0.1% Tween 20 in PBS. After incubation with either an anti-mouse or anti-rabbit HRP-labeled secondary antibody (GE Healthcare) for 1 h at room temperature, blots were developed using enhanced chemiluminescence detection Western blotting reagents (SuperSignal West Pico Chemiluminescent Substrate, Pierce, Thermo Scientific). To determine the level of basal pERK level, which was found to be low in PC12 cells, we used both long exposure of blots to the above-mentioned reagent and ECL PLUS (Amersham) in order to enhance chemiluminescent signals. For the normalization of data, membranes were incubated in buffer (65 mM Tris, pH 6.8, 2% SDS, *w*/*v*, β-mercaptoethanol) to strip the previous antibodies, and re-probed with antibodies for α-tubulin (1:2000, Sigma), total ERK (1:1000, Cell Signaling) or Akt(pan) (1:1000, Cell Signaling). The optical densities of the positive bands of the scanned films were quantified using NIH Image Analysis software version 1.43.

### 3.7. Statistical Analysis

Data are presented as the means ± SEM. If three or more groups of data were compared, the statistical significance of differences was assessed by one-way analysis of variance (ANOVA) followed by Tukey’s *post hoc* test for multiple comparisons. Student’s *t* test was used to characterize the significance of differences between two groups of data. The differences were considered significant at *p* < 0.05.

## 4. Conclusion

The present work is one of the first attempts at studying the protective effect of α-T at nanomolar concentration, which are its physiological concentrations in cerebrospinal fluid and brain extracellular space. Pre-incubation of PC12 cells with α-T at nanomolar concentrations for 3–18 h was found to protect PC12 neuronal cell line from H_2_O_2_-induced cell death. The protective effect of α-T against H_2_O_2_ toxicity is concentration-dependent in the nanomolar range (1 nM < 10 nM < 100 nM) if pre-incubation with it was performed for 18 h. Under such conditions of experiments, the maximal protective effect could be achieved by 100 nM α-T. A further increase in its concentration (1 μM, 10 μM and 100 μM) did not result in higher protection. Using flow cytometry α-T at the nanomolar concentration was shown for the first time to have anti-apoptotic activity in cells of the neuronal cell line under conditions of oxidative stress. Such an effect of nanomolar α-T has only been observed in the case of long pre-incubation and was absent if pre-incubation was performed for 0.5 h. Nanomolar α-T was found to protect cultured immature cortical neurons from H_2_O_2_-induced death if pre-incubation was performed for 18 h, but it was not effective if pre-incubation was performed for 0.5 h. However, the effect of micromolar α-T is pronounced at any time of pre-incubation.

We studied the possible contribution of modulation of ERK 1/2, Akt and PKC activity to the protective effect of nanomolar and micromolar α-T on PC12 cells exposed to hydrogen peroxide. The data obtained suggest that the ability of α-T to reduce the time of maximal activation of ERK 1/2 and Akt contributes to its protective effect, as well as the modulation of PKC activity in PC12 cells exposed to H_2_O_2_. However, the protective effect of α-T may also depend on other signaling pathways.

## Figures and Tables

**Table 1:** The protective effect of α-T against hydrogen peroxide-induced PC12 cell death depends on the time of pre-incubation (rescue rates of α-T, %).

Pre-incubation Time/Sample	3 h	6 h	12 h	18 h

	Rescue Rates %			
100 μM α-T	67.7 ± 3.95 *,^x^	72.6 ± 4.6 *,^x^	67.1 ± 3.3 *	66.2 ± 4.5 *
100 nM α-T	44.0 ± 6.9 *,^x^	48.2 ± 5.8 *,^x^	61.2 ± 3.6 *^x^	63.9 ± 3.5 *,^x^
10 nM α-T	10.5 ± 2.4 *	24.2 ± 1.2 *	28.9 ± 5.4 *,#	33.1 ± 2.4 *,#
1 nM α-T	2.90 ± 3.8	7.2 ± 4.1	10.5 ± 0.5 *	15.8 ± 0.4 *
0.1 nM α-T	1.3 ± 3.2	2.3 ± 5.08	1.6 ± 7.1	6.9 ± 7.0

* the protective effect of α-T is significant, *p* < 0.05;

x,# the differences are significant as compared to the effect of α-T in all lower concentrations (for the same time of pre-incubation) according to one-way ANOVA followed by Tukey’s multiple comparison test, ^x^ *p* < 0.02, ^#^ *p* < 0.05.

**Table 2:** The table shows that the protective effect of α-T against H_2_O_2_-induced PC12 cell death is diminished or abolished in the presence of inhibitors of PKC, MEK 1/2 and PI 3-kinase if pre-incubation time with α-T is 18 h prior to PC12 cell exposure to 0.2 mM H_2_O_2_ for 24 h. Pre-incubation of PC12 cells with protein kinase inhibitors was performed for 0.5 h before addition of α-T to the incubation medium.

Sample	Rescue Rates of α-T, %
100 nM α-T	53.3 ± 4.9 *
100 nM α-T + 1 μM GF109203X	20.7 ± 8.0 x
100 nM α-T	48.3 ± 6.0 *
100 nM α-T + 10 μM SL327	34.5 ± 4.9 *,#
100 nM α-T	44.9 ± 6.6 *
100 nM α-T + 50 μM LY294002	19.8 ± 10.1 #
100 nM α-T	53.7 ± 5.4 *
100 nM α-T + 10 μM LY294002	48.2 ± 2.1 *
100 nM α-T	52.2 ± 6.3 *
100 nM α-T + 10 μM SL327 + 10 μM LY294002	39.7 ± 8.3 *,^x^
100 μM α-T	55.9 ± 6.9 *
100 μM α-T + 1 μM GF109203X	19.7 ± 9.0 x
100 μM α-T	55.7 ± 6.2 *
100 μM α-T + 10 μM SL327	36.5 ± 5.2 *,#
100 μM α-T	53.4 ± 9.5 *
100 μM α-T + 50 μM LY294002	29.8 ± 6.9 *,#
100 μM α-T	52.6 ± 5.7 *
100 μM α-T + 10 μM LY294002	48.3 ± 4.5 *
100 μM α-T	57.7 ± 7.6 *
100 μM α-T + 10 μM SL327 + 10 μM LY294002	39.3 ± 7.4 *,^x^

The data are expressed as rescue rates of α-T, %. The data are means ± SEM from 4 to 6 experiments. The cell viability was assessed by the LDH method. In this table:

* the protective effect of α-T is significant, *p* < 0.05;

x,# the differences are significant as compared to the effect of α-T in the absence of inhibitors by paired Student’s *t* test; ^x^ *p* < 0.02; ^#^ *p* < 0.05.

## Abbreviations

α-T: α-tocopherol
ERK 1/2: extracellular signal-regulated kinase 1/2
MEK 1/2: extracellular signal-regulated kinase kinase 1/2
Akt: protein kinase B
PI 3-kinase: phosphoinositide 3-kinase
PKC: protein kinase C
